# The administration of immune checkpoint inhibitors via an elastomeric pump versus conventional intravenous infusion: an economic perspective

**DOI:** 10.1186/s12913-024-11719-0

**Published:** 2024-10-31

**Authors:** Michiel Zietse, Ruben Malmberg, Roelof W.F. van Leeuwen, Frederick W. Thielen, Carin A. Uyl – de Groot

**Affiliations:** 1 Department of Hospital Pharmacy, Erasmus University Medical Center, Rotterdam, the Netherlands; 2 Department of Medical Oncology, Erasmus University Medical Center, Rotterdam, Netherlands; 3 Department of Health Technology Assessment, Erasmus School of Health Policy and Management, Erasmus University Rotterdam, Rotterdam, The Netherlands; 4 Erasmus Centre for Health Economics Rotterdam (EsCHER), Erasmus University Rotterdam, Rotterdam, The Netherlands

**Keywords:** Immune checkpoint inhibitors, Elastomeric pump, Intravenous infusion, Efficiency, Micro costing, Nivolumab, Pembrolizumab

## Abstract

**Background:**

Recent studies have underscored the potential of innovative administration methods to mitigate the capacity burden on healthcare systems, without compromising the quality of care. This study assessed and compared the resource utilization and associated costs of two distinct administration modes of immune checkpoint inhibitors: the innovative elastomeric pump and conventional intravenous infusion. This comparison can inform sustainable healthcare practices and healthcare decision-making to optimize treatment efficiency in an era of escalating healthcare demands.

**Methods:**

In this micro-costing study, data on resource use and time allocation for drug preparation and administration were collected using an observational, non-interventional study design. Data were registered at the oncology daycare unit and hospital pharmacy. Cost categories included drug acquisition, disposable materials, healthcare professional time for drug administration, drug preparation, and patient time spent at the oncology day care unit.

**Results:**

Drug administration through the elastomeric pump resulted in substantially lower healthcare costs when compared to conventional infusion, particularly due to reduced labor and chair time. The elastomeric pump reduced the total chair time by 78% and nurse time by 55%. Total average costs (excluding drug costs) were €103,47 and €77.99 for conventional infusion and the elastomeric pump, respectively, showcasing potential savings of €25.48 (*P* < 0.001) per administration.

**Conclusions:**

This study demonstrated that the elastomeric pump not only offers substantial cost savings but also enhances the treatment capacity of the oncology day care unit. These findings support the adoption of the elastomeric pump in clinical settings as a cost-saving and efficient alternative to conventional infusion.

**Trial registration:**

This study has been registered in the National Trial Register (NTR), with the reference number NTR NL9473. Registration date: 05-05-2021.

**Supplementary Information:**

The online version contains supplementary material available at 10.1186/s12913-024-11719-0.

## Background

The introduction of immune checkpoint inhibitors (ICIs), such as nivolumab and pembrolizumab, has revolutionized cancer treatment by offering durable responses to patients with solid and hematological cancers. As such, ICIs have become standard therapy in numerous tumor types and settings. With the development of promising combination treatment approaches, it is expected that ICIs will continue to be fundamental in treating an expanding range of malignancies in the future [1].

Despite the evident advantages, the widespread application of ICIs presents novel challenges. The rising prevalence of cancer, combined with the expanded regulatory approval of ICIs has led to a swift increase in the patient population receiving ICI therapy. ICIs are conventionally delivered via an IV bag (ICI-B) over a thirty-minute to one-hour infusion at the oncology day care unit, confining patients to an infusion chair. Given the repetitive nature of these treatments, often spanning years, it places considerable pressure on the limited resources, particularly the available chairs at outpatient oncology clinics and the nursing workforce [2]. Presently, many countries are facing oncology nurse staffing shortages which could potentially worsen in the future [3]. This, combined with a substantial increase in demand for cancer care services, including ICI infusion, could lead to future treatment delays [4].

Moreover, the costs associated with the time spent at the oncology day care unit (chair time) have also been shown to be drivers of the total treatment costs [5]. Amongst other expenses, these include the costs of labor and overhead. As a result, reducing chair time has proven to be an effective strategy for addressing capacity challenges and reducing drug administration costs. The feasibility of this approach has been previously demonstrated with subcutaneous (SC) formulations of monoclonal antibodies (mAbs) [6, 7].

Considering such strategies, the administration of ICIs via an elastomeric pump (ICI-P) holds significant promise. Currently, the feasibility and patient preference for ICI-P is investigated in an ongoing clinical trial (Ref: NTR NL9473) [8]. Elastomeric pumps are portable, non-mechanized, self-deflating infusion devices for fluids at a specified rate. Notably, the dosage used in ICI-P is consistent with that in ICI-B. Elastomeric pumps are increasingly being used to facilitate outpatient treatment and enable early hospital discharge, continuing intravenous (IV) treatment at home (e.g. antibiotics) [9].

During ICI-P infusion, patients are free to move unrestrictedly within the hospital premises. This flexibility allows them to visit various hospital facilities, including the restaurant, during their infusion. Once the infusion is completed, patients return to the oncology day care unit, where an oncology nurse disconnects the elastomeric pump prior to their discharge. While the patients receive their infusion, the treatment chair remains unoccupied, presenting an opportunity to potentially treat other patients. By minimizing the chair time required per treatment cycle, the oncology day care unit can increase its capacity and throughput.

To date, the healthcare costs of this novel administration strategy compared with conventional infusion have not been studied. Therefore, this research aims to determine and compare the time and costs associated with the preparation and administration of ICI-P compared with ICI-B within a cost-minimization framework. By elucidating the potential benefits and efficiencies of ICI-P, this study aims to equip healthcare providers, policymakers, insurers, patients, and pharmaceutical companies with pivotal insights, fostering informed decision-making and optimizing healthcare resource use in clinical practice.

## Methods

### Study design

An observational, non-interventional, bottom-up micro costing study was performed to enable a cost-minimization analysis. The focus was on the healthcare costs of ICI-P and ICI-B from a hospital perspective. This micro costing study was conducted on a prospective basis alongside and as part of the ongoing clinical trial (Ref: NTR NL9473) in which it is the primary objective to establish the patient preference for either ICI-B or ICI-P [8].

Eligible patients were over 18 years old with any standard of care indication for nivolumab or pembrolizumab monotherapy at any EMA-approved dose. All participants had completed at least three cycles of ICI treatment prior to trial enrollment. Patients who had previously experienced an infusion-related reaction (IRR) according to Common Terminology Criteria for Adverse Events version 5.0 (CTCAE v5.0) (any grade) during ICI treatment were excluded from participation in this study. Participants in the ICI-P group were allowed to leave the oncology day care unit during infusion.

The study was approved by the medical ethics board of the Erasmus University Medical Center, Rotterdam, The Netherlands (reference number: MEC-2021-0250) and was conducted in accordance with the Declaration of Helsinki. All included subjects gave informed consent.

### Data collection

**Fig. 1:**
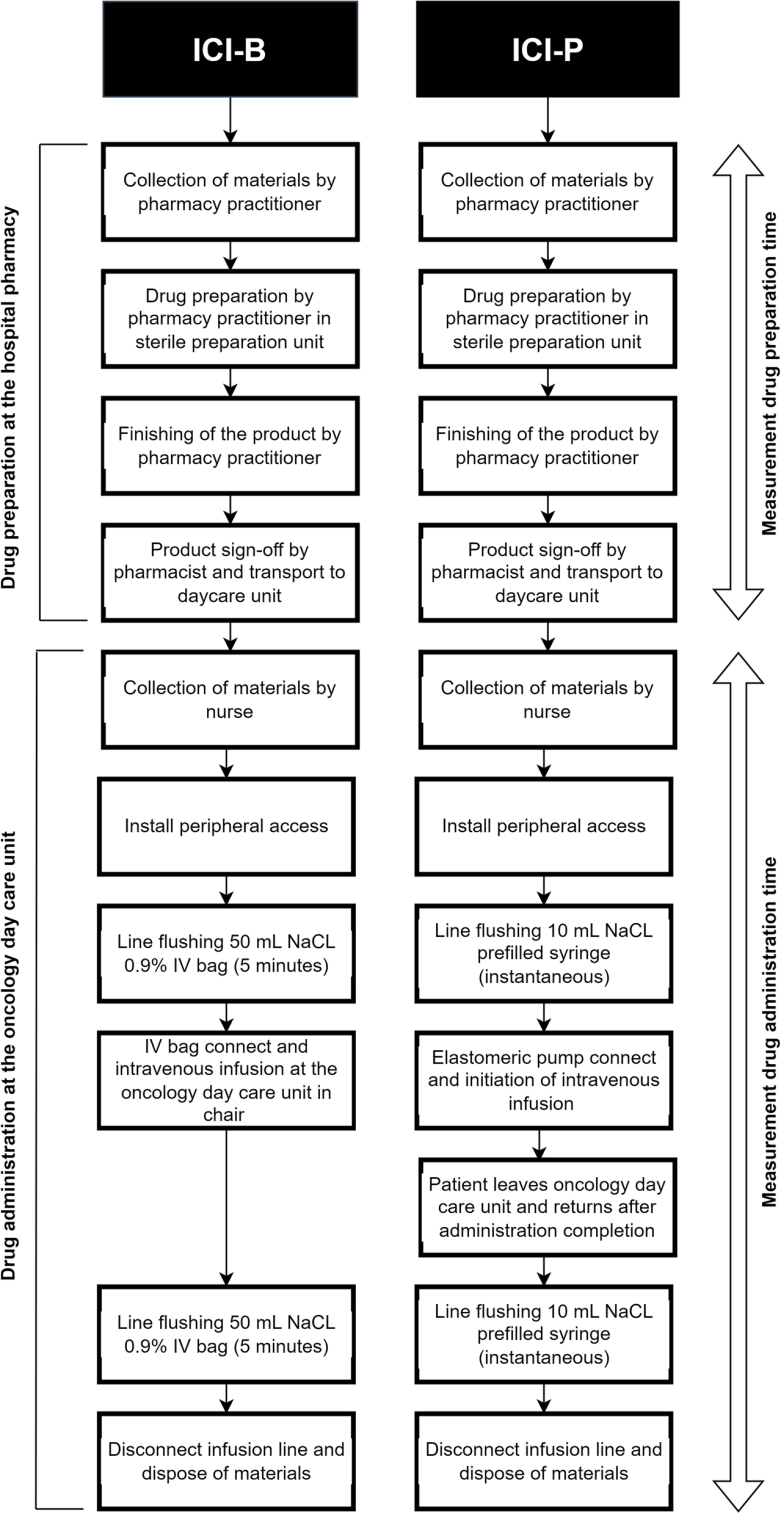
Procedures of ICI-B and ICI-P drug preparation and drug administration. ICI-B indicates conventional infusion of immune checkpoint inhibitors; ICI-P, immune checkpoint inhibitors administrated via an elastomeric pump

Data on the total resource use and time allocation for drug preparation and administration were collected at the hospital pharmacy and oncology day care unit of the Erasmus University Medical Center, using standardized case report forms (CRF). To prevent the overrepresentation of individual patients in the analysis, a maximum of two observations per participating patient was permitted. HCPs could participate in multiple observations, with the assumption that all were similarly efficient. The procedures of the drug preparation and drug administration of ICI-B and ICI-P are shown in Fig. 1.

The drug preparation process consisted of four distinct steps, for which stopwatch time measurements (seconds and minutes) were collected: (1) Collection of the materials (2) Preparation of ICI-B or ICI-P (3) Finishing of the product (4) Product sign-off. Steps 1–3 were performed by pharmacy technicians and step 4 by a (hospital) pharmacist. Time of transport was not included as the drugs were transported via a mechanized tubular transport system that directly connects the hospital pharmacy with the oncology day care unit.

The drug administration CRF included relevant time measurements to calculate the total chair time and active healthcare professional (HCP) time. Total chair time was based on the time spent at the oncology day care unit during treatment. Stopwatch time measurements were used to determine the active HCP time, including tasks such as material collection, peripheral access installation, line flushing, device connection, line disconnection, and patient-related tasks (e.g., explanations and conversations).

### Resource valuation

Costs were calculated by multiplying resource use volumes by standard unit prices in Euro, following the methodology outlined in the Dutch costing manual [10, 11]. Prices were adjusted to 2022 values using the general consumer price index obtained from the Dutch Central Bureau of Statistics [12]. The cost categories included costs of drugs, disposable materials, healthcare professional wages, and the time spent at the oncology day care unit. Drug costs were based on the Dutch standard list prices (2022) [13]. To ensure uniformity in healthcare costs for ICI-B and ICI-P, drug costs were standardized based on the registered fixed doses of six-weekly 400 mg pembrolizumab and four-weekly 480 mg nivolumab. This standardization was necessary since drug expenses accounted for a significant portion of the drug cost and were independent of the administration method, considering dosing is identical for ICI-P and ICI-B.

To compute the total costs of disposables, the volume of resource use was multiplied by the costs per disposable (2022), which were obtained from the hospital’s financial administration. The costs of HCP time were determined by multiplying the observed active HCP time with the respective wage rates of nurses, pharmacy technicians, and pharmacists. These wage rates were obtained from the hospital’s financial administration. For the costs of time spent at the oncology day care unit, overhead costs were considered based on the methods outlined by Franken et al. (2018) [6]. Overhead costs were computed using the annual cost of the oncology day care unit minus the costs of nurses, drugs and consumables, divided by the annual number of admissions. The average costs per admission were divided by the average admission duration, resulting in the costs per minute spent at the oncology day care unit, which were multiplied by the actual time spent at the oncology day care unit for each patient. Table 1 provides an overview of all unit costs employed. Additional information regarding the quantity and types of disposables used for the preparation and administration of ICI-B and ICI-P can be found in the Supplementary Appendix.

**Table 1:** An overview of the unit costs in euros (2022 prices)

Price per item (Euro 2022 prices)	
**Time of healthcare professionals**	
Pharmacist (per minute)	€0.95
Nurse (per minute)	€0.67
Pharmacy assistant (per minute)	€0.48
**Outpatient oncology daycare unit**	
Daycare unit overhead (per minute)	€0.97
**Drug costs**	
Nivolumab 40 mg	€405.03
Nivolumab 100 mg	€1,012.56
Nivolumab 240 mg	€2,430.15
Pembrolizumab 100 mg	€2,591.18
**Disposables drug preparation (per item)**	
Intermate SV 100 105mL (Elastomeric pump)	€43.60
Infusion bag sodium chloride 0.9% 50 ml	€0.42
Infusion bag sodium chloride 0.9% 100 ml	€0.42
Syringe 50 mL	€0.35
Blunt fill needle	€0.04
Alcohol wipe	€0.57
Isopropylalcohol wipe	€0.02
Non-woven compress (sterile) 10 × 10	€0.13
Sterile pair of gloves	€0.63
Mask	€0.09
Hat	€0.12
Shoe-covers	€0.15
Minigrip bag	€0.14
**Disposables drug administration (per item)**	
Chlorhexidine tincture	€1.60
Connect set	€0.81
Adhesive bandages	€2.02
Face mask	€0.09
I.V. administration set (ProSet Infusomat)	€2.34
Infusion bag sodium chloride 0.9% 250 mL	€0.42
Infusion filter	€2.88
IV Cannula	€2.83
Nonsterile gauze (5 × 5)	€0.03
Nonsterile gloves	€0.12
Adhesive Cannula dressing	€0.70
Transfersystem with spike adapter	€3.11

### Statistical analysis

Descriptive statistics (averages, medians, and interquartile ranges (IQR)) were used for time measurements, resource use, and costs. Non-parametric bootstrapping was employed to analyze the statistical significance of differences in the total costs, costs of HCP time, resource use, and chair time. The independent-samples T-test with bootstrap estimates was used for this purpose. The statistical analysis was conducted using IBM SPSS Statistics, version 28.0.1.0. To evaluate uncertainty surrounding cost estimates and their impact on the difference in healthcare costs between ICI-P and ICI-B, a one-way deterministic sensitivity analysis was performed by varying the time measurements and resource utilization to the most extreme values of the observation data or input values obtained from the literature. The exhaustive list of parameters considered includes the price of the elastomeric pump, costs per minute spent at the day care unit, minutes of chair time, active HCP time, and HCP wage costs.

## Results

### Disposable costs and drug costs

Table 2 illustrates the costs associated with the disposables required for the administration of ICI-B and ICI-P. The drug costs were equal for both administration methods. In terms of disposable costs alone, ICI-P had the highest total costs of €50.62, mainly driven by the price of the elastomeric pump. ICI-B had costs of €16.15 and €13.27 for nivolumab and pembrolizumab, respectively, which differed due to the infusion line filter used only for nivolumab via ICI-B administration. As a result, the average disposable cost for ICI-P exceeded those of ICI-B by €37.35. Drug costs were equal for ICI-B and ICI-P, which were €4,860.30 and €10,497.52 for nivolumab and pembrolizumab, respectively.

**Table 2:** Disposable and drug costs per administration of ICI-B and ICI-P. ICI-B indicates conventional infusion of immune checkpoint inhibitors; ICI-P, immune checkpoint inhibitors administrated via an elastomeric pump

	Nivolumab 480 mg			Pembrolizumab 400 mg		
	ICI-B	ICI-*P*	Incremental	ICI-B	ICI-*P*	Incremental
*Drug preparation disposables*						
Elastomeric pump	**-**	€43.60	€43.60	**-**	€43.60	€43.60
Sodium chloride	€0.42	€0.42	**-**	€0.42	€0.42	
Syringe and needles	€0.43	€0.78	€0.35	€0.78	€0.78	€0.35
Protective materials (e.g., sterile gloves)	€0.99	€0.99	**-**	€0.99	€0.99	**-**
Cleaning materials (e.g., isopropyl alcohol wipes)	€0.65	€0.65	**-**	€0.65	€0.65	**-**
**Total drug preparation disposables**	**€2.76**	**€46.71**	**€15.75**	**€2.76**	**€18.51**	**€15.75**
*Drug administration disposables*						
IV infusion related (e.g., infusion line)	€11.16	€2.83	€8.33	€8.28	€2.83	€5.45
Protective materials (e.g., nonsterile gloves)	€0.21	€0.21	-	€0.21	€0.21	**-**
Sodium chloride	€0.42	€0.08	€0.34	€0.42	€0.08	€0.34
Others (e.g., disinfectant, bandages)	€1.60	€1.60	**-**	€1.60	€1.60	**-**
**Total drug administration disposables**	**€13.39**	**€3.91**	**€9.48**	**€10.51**	**€3.91**	**€6.60**
**Total disposables costs**	**€16.15**	**€50.62**	**€34.47**	**€13.27**	**€50.62**	**€37.35**

### Time resource use of drug administration and preparation

The observational study included 16 participants and involved 22 observations of drug administration and 32 observations of drug preparation collected between March and May 2022. Table 3 shows the allocated time utilization for ICI-B and ICI-P. As anticipated, the average time patients spent at the oncology day care unit was substantially longer for ICI-B compared with ICI-P (74.50 vs. 16.25 min). During infusion, patients receiving ICI-P spent on average 59 min outside the oncology day care unit. Notably, the average infusion duration of ICI-P was 23% longer than ICI-B (60.83 vs. 49.40 min). The overall treatment duration for ICI-P was similar to that of ICI-B.

**Table 3:** Time measurements of active HCP time during drug preparation and administration, and time spent at the day care unit. ICI-B indicates conventional infusion of immune checkpoint inhibitors; ICI-P, immune checkpoint inhibitors administrated via an elastomeric pump

HCP Time required per task and patient time spent at the day care unit (minutes)								
	**ICI-B** *(Preparation n = 22, Administration n = 10)*				**ICI-P** *(Preparation n = 10, administration n = 12)*			**Incremental time (ICI-B minus ICI-P)**
	Average	Median	IQR	Average		Median	IQR	Difference
*Drug preparation*								
Collecting materials	2.23	2.18	1.83–2.60	2.13		2.02	1.92–2.57	0.10
Preparing drug	1.88	1.73	1.51–2.03	6.76		5.17	4.52–6.08	-4.88
Finishing product	0.93	0.90	0.60–1.10	1.43		1.18	1.07–1.33	-0.50
Product sign-off	0.39	0.30	0.20–0.50	0.49		0.38	0.18–0.76	-0.09
**Total drug preparation active pharmacy technician time**	**4.94**	**4.53**	**4.11–5.56**	**9.68**		**8.25**	**6.67–9.60**	**-4.74**
**Total drug preparation active pharmacist time**	**0.39**	**0.30**	**0.20–0.50**	**0.49**		**0.38**	**0.18–0.76**	**-0.09**
*Drug administration*								
Collecting of materials	3.48	3.08	2.33–2.80	3.18		2.99	2.31–3.98	0.30
Install of intravenous access	4.39	4.61	2.18–5.89	5.07		4.02	2.53–5.94	-0.69
Pre-administration flushing	5.85	5.03	4.91–5.81	-		-	-	5.85
Post-administration flushing	5.63	5.72	5.19–5.95	-		-	-	5.63
Disconnect and dispose of materials	1.83	1.98	1.50–2.20	1.79		1.79	1.48–2.07	0.05
Time spent with patient beyond standard tasks	0.90	0.00	0.00–0.00	0.00		0.00	0.00–0.00	0.90
**Total drug administration active nurse time**	**22.07**	**21.46**	**19.78–24.45**	**9.62**		**8.25**	**7.69–11.10**	**12.45**
*Patient time*								
Infusion time	49.40	51.00	45.50-55.25	60.83		60.50	58.75–63.25	-11.43
Time spent outside day care unit	-	-	51.50-60.25	56.25		59.00	51.50-60.25	56.25
**Total treatment duration**	**73.30**	**74.50**	**68.50–77.00**	**72.50**		**69.00**	**66.00–77.00**	**0.80**
**Total chair time**	**73.30**	**74.50**	**68.50–77.00**	**16.25**		**15.50**	**13.00-18.50**	**57.05**

Despite the similar total treatment durations, ICI-P successfully reduced the average active nursing time by 12 min compared to ICI-B. The most substantial savings of nursing time were observed for the pre-and post-hydration procedures during ICI-B, which were not required during the administration procedure of ICI-P and amounted to 11 min on average. Nurses also spent more time on non-standard tasks with the patient during ICI-B treatment (0.9 min on average). In contrast, drug preparation of ICI-P demanded almost twice the time of pharmacy technicians compared with ICI-B (9.68 vs. 4.94 min on average). The largest difference was observed in the preparation step, which required 1.88 min for ICI-B, compared with 6.76 min for ICI-P.

### Healthcare costs of ICI-B and ICI-P

Table 4 presents the total healthcare costs of ICI-B and ICI-P. Total average costs were €103,47 and €77.99 for ICI-B and ICI-P, respectively, showcasing potential savings of €25.48 (< 0.001) for ICI-P. The costs of time spent at the oncology day care unit were the largest cost item for ICI-B, with average costs of €72.38 compared with €15.79 for ICI-P. Disposable costs, in particular the costs of the elastomeric pump, were responsible for the largest costs of ICI-P (€50.62).

**Table 4:** Valuation of ICI-B and ICI-P resource use in euros. ICI-B indicates conventional infusion of immune checkpoint inhibitors; ICI-P, immune checkpoint inhibitors administrated via an elastomeric pump

Resource use valuation (€)								
	ICI-B*			ICI-*P*			Incremental costs (ICI-B minus ICI-*P*)	
	Average	Median	IQR	Average	Median	IQR	Difference	P-value
*Costs of healthcare professionals*								
Pharmacy technician	€2.39	€2.19	€1.99-€2.69	€4.68	€3.99	€3.22-€4.64	-€2.29	-
Pharmacist	€0.37	€0.29	€0.19-€0.48	€0.46	€0.36	€0.17-€0.72	-€0.09	-
Nurse	€14.78	€14.37	€13.25-€16.38	€6.44	€5.53	€5.15-€7.43	€8.34	-
**Total costs of healthcare professionals**	**€17.55**	**€16.85**	€**15.43-**€**19.54**	**€11.58**	**€9.87**	**€8.54-€12.79**	**€5.96**	**< 0.001**
*Costs of disposables*								
Drug preparation disposables	€2.76	-	-	€46.71	-	-	-€15.75	-
Drug administration disposables	€11.95	**-**	**-**	€3.91	-	-	€9.48	-
**Total costs of disposables**	**€17.55**	**€16.85**	€**15.43-**€**19.54**	**€11.58**	**€9.87**	**€8.54-€12.79**	**€5.96**	**< 0.001**
Costs of time spent at the day care unit	€71.22	€72.38	€66.55-€74.81	€15.79	€15.06	€12.63-€17.97	€55.43	< 0.001
**Total healthcare costs (excluding drug costs)**	**€103.47**	**€103.94**	**€96.69-€109.06**	**€77.99**	**€75.55**	**€71.79-€81.39**	**€25.48**	**< 0.001**

Regarding the total labor costs, ICI-P achieved average savings of €5.96 compared to ICI-B (*P*-value < 0.001), even though the costs of labor during drug preparation were higher for ICI-P compared to ICI-B. This was primarily due to the lower costs associated with active nursing time during ICI-P treatment. Specifically, the average labor costs during drug administration were €14.78 for ICI-B and €6.44 for ICI-P which offset the higher labor costs during ICI-P preparation by pharmacy technicians.

### Sensitivity analysis

**Fig. 2:**
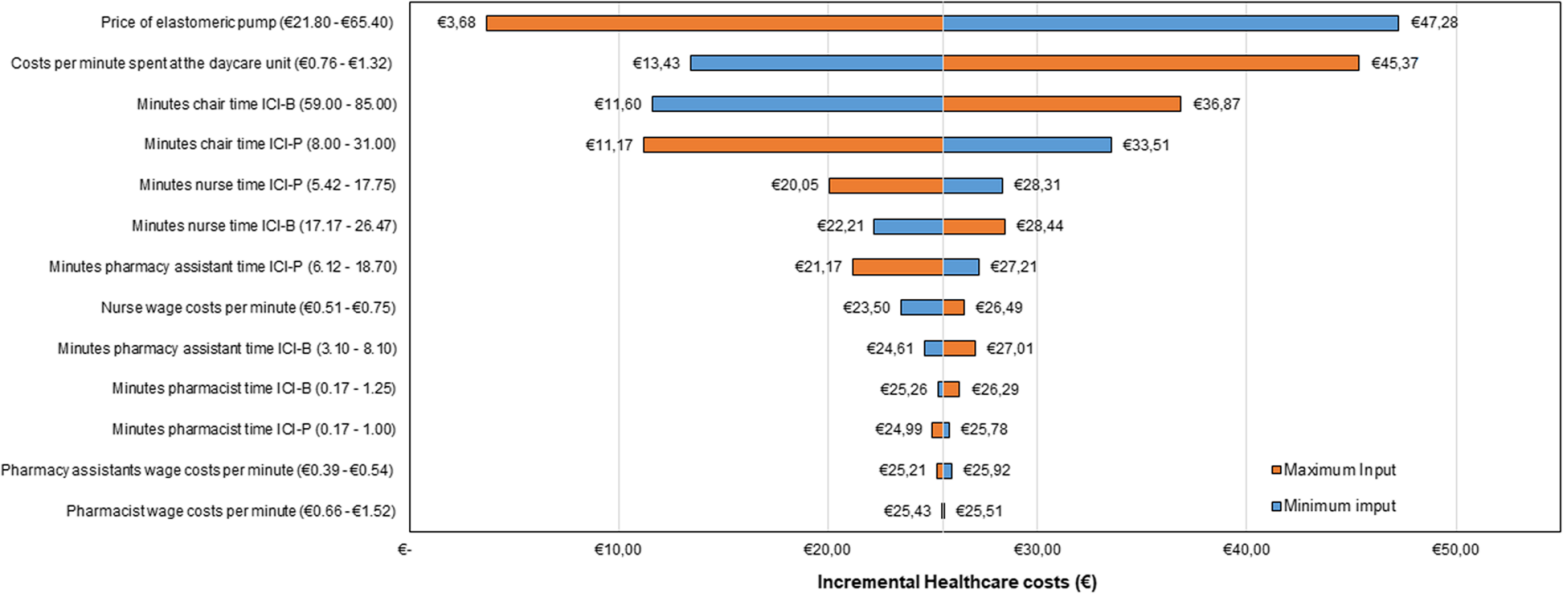
Tornado diagram of the incremental healthcare cost estimates obtained from the one-way deterministic sensitivity analysis. A positive incremental value indicates that ICI-P is cheaper compared to ICI-B. Estimates of minimum input values are in blue and estimates of maximum input values in orange. ICI-B indicates conventional infusion of immune checkpoint inhibitors; ICI-P, immune checkpoint inhibitors administrated via an elastomeric pump

Figure 2 presents the results of the one-way deterministic scenario analysis. The analysis consistently demonstrates that ICI-P’s healthcare costs are lower than those associated with ICI-B, resulting in positive incremental healthcare costs. The healthcare costs and potential savings were especially sensitive to changes in input parameters related to the price of the elastomeric pump, costs of the time spent at the oncology day care unit, and the total chair time during ICI-B and ICI-P treatment. Particularly, the price of the elastomeric pump had the most substantial impact on the incremental healthcare costs, ranging from €3.68 to €47.28. This indicates that variations in the elastomeric pump’s price greatly influence the overall cost difference between ICI-P and ICI-B. Furthermore, the total chair time for ICI-P and ICI-B exhibited an inverse relationship. Increasing the total chair time of ICI-P resulted in lower potential savings while increasing the chair time of ICI-B enhanced the potential savings of ICI-P.

Additionally, the active nursing time during ICI-P treatment had a greater influence on the potential savings compared with the active time of pharmacists or pharmacy technicians. Generally, the wage costs of healthcare professionals contributed only marginally to the incremental healthcare cost, with nurse wage costs creating the most significant impact, while pharmacy technicians and pharmacist wage costs had a negligible effect (< 1%).

## Discussion

Our study compared the healthcare costs of ICI-P and ICI-B, revealing that ICI-P resulted in substantial reductions in chair occupancy and nursing time and was therefore a cheaper and more efficient infusion method. These findings support the adoption of the elastomeric pump in clinical settings as a cost-saving and efficient alternative to conventional infusion.

Our analysis demonstrated that ICI-P could reduce the total chair time by 78% and nurse time by 55%, aligning with potential savings reported in prior studies when switching from IV to SC anticancer mAbs [6, 7, 14, 15]. Similarly, the potential savings of SC administration were mostly due to reduced time spent at the oncology day care unit and wage costs. In a recent systematic review, McCloskey et al. (2023) reported the active time of nurses ranging from 17 to 35 min during IV administration of rituximab. Although this study observed different drugs, these data are comparable to the findings in this study (22 min of average active nurse time during ICI-B administration) [16].

Currently, there are no marketed SC formulations of nivolumab and pembrolizumab, making ICI-P a viable alternative to ICI-B. While these subcutaneous formulations are being investigated (NCT03656718, NCT03665597) [17, 18], their fixed dosages present challenges concerning dosing flexibility and potential cost-saving strategies [19]. The fixed dosages for the subcutaneous forms of nivolumab and pembrolizumab are projected to exceed those of their intravenous counterparts [20–22]. Given that drug costs significantly outweigh the expenses of preparation and administration, this aspect demands careful consideration. Additionally, while SC administration could enable at-home treatment, improving patient convenience, it may result in higher costs and reduced efficiency outside of hospital settings, primarily due to the significant time commitments required from nurses [23]. 

The majority of the potential savings of ICI-P were associated with a reduction in fixed costs, notably overhead expenses and hospital staff wages. Yet, these theoretical savings may not materialize into actual savings unless hospitals effectively reduce their labor and overhead costs. However, the concept of opportunity costing should also be considered [15]. With ICI-P requiring significantly less chair time compared to ICI-B, approximately 4.5 patients can be treated with ICI-P for each ICI-B patient based on chair time. This substantial increase in chair turnover allows for higher patient throughput and reduces overhead costs per patient. Furthermore, reducing the active time of pharmacy staff and nurses allows them to potentially transition to other tasks, increasing their overall productivity. Consequently, it is justifiable to include these fixed-cost items in the computation of the total healthcare costs.

Contrarily, the disposable costs of ICI-P were found to be considerably higher due to the elastomeric pump’s pricing. However, it should be noted that this analysis utilized the pump’s list price, and most hospitals often receive substantial discounts on disposables. As the overall costs are significantly sensitive to the pump’s price, potential real-world savings may surpass those suggested by our findings.

This study is the first to investigate the healthcare costs related to ICI-P administration, employing a bottom-up micro costing approach. This approach considered the gold standard methodology in costing studies, provided more precise estimations compared to other costing methods [24].

A limitation of this study is the modest sample size, which could imply that outlier data might have disproportionately influenced our findings. However, due to the large degree of standardization within the drug preparation and drug administration procedures, the variation within the time measurements remained limited.

Further, our data was collected from a single and large hospital in the Netherlands, potentially limiting the generalizability of the results due to scale advantages. This could lead to a possible underestimation of drug preparation costs in smaller hospitals. McCloskey et al. (2023) reported IV preparation times ranging between 14 and 21 min, substantially longer than the preparation of ICI-B which took only 5 min on average in this study [16]. However, it is important to note that these studies focused on IV trastuzumab preparation, a drug that, unlike nivolumab and pembrolizumab, requires reconstitution, thereby consuming more time for pharmacy technicians [25]. Moreover, the costs of drug preparation constitute only a limited portion of the total healthcare costs associated with ICI-B and ICI-P.

Another potential concern with ICI-P is the possible decrease in quality interaction time between patients and HCPs. However, our findings challenge this notion as only in one instance of ICI-B administration the nurse spent additional time with the patient. All other conversations with the patient took place while concurrently executing other treatment administration-related tasks. This suggests that ICI-P might not significantly impact the patient’s perceived quality of care based on interaction with HCPs. However, these observations may not be universal and could be influenced by the working culture and pressure within different hospitals.

Furthermore, the applicability of ICI-P remains uncertain. For example, some patients might be ineligible for ICI-P due to previous infusion reactions or reluctance to leave the oncology day care unit during treatment. Also, the feasibility of ICI-P in other hospitals and countries is yet to be determined. Moreover, the feasibility of implementing the ICI-P model depends on the specific hospital setting, as it requires not only adequate space but also appropriate facilities where patients can comfortably spend time during their infusion.

Despite these possible limitations, the potential costs and nurse time savings of ICI-P are considerable. In addition to monetary savings, ICI-P could increase treatment capacity and enhance nursing productivity. In addition, the use of elastomeric pumps could be expanded to other drugs (e.g., with longer administration times) to further increase the financial and capacity benefits.

As cost and capacity savings should not compromise the quality of care, safety and patient-reported outcomes such as treatment satisfaction and preference should be prioritized when evaluating the implementation of ICI-P in clinical practice. Accordingly, we await the final analysis of the parent study before recommending ICI-P as a standard of care method for ICI infusion.

## Conclusions

This study demonstrated that ICI-P not only offers substantial cost savings but also enhances the treatment capacity of the oncology day care unit. Although there were possible limitations to the study, including sample size and generalizability, the results provided valuable insights into the economic implications of ICI-P administration. The results in this study may also be extrapolated to other (cytostatic) drugs. Further research is warranted to validate and expand these findings to routine clinical practice.

## Supplementary Information

Supplementary Material 1.

## Data Availability

All data analyzed during this work are from the Erasmus University Medical Center (EMC), Rotterdam. The data are not publicly available, but can be requested from EMC.

## Abbreviations

CRF: Case report form
CTCAE v5.0: Common Terminology Criteria for Adverse Events version 5.0
HCP: Healthcare Professional
ICI-B: Administration of immune checkpoint inhibitors via an IV bag
ICI-P: Administration of immune checkpoint inhibitors via an elastomeric pump
ICIs: Immune checkpoint inhibitors
IRR: Infusion-related reaction
IQR: Interquartile range
IV: Intravenous
SC: Subcutaneous
