# Supplementary material for: The administration of immune checkpoint inhibitors via an elastomeric pump versus conventional intravenous infusion: an economic perspective
Source: BMC Health Serv Res. 2024 Oct 31;24:1322. doi: 10.1186/s12913-024-11719-0 (PMC11526519; doi:10.1186/s12913-024-11719-0)
Supplement: Supplementary file 1 — Supplementary Material 1. [file 12913_2024_11719_MOESM1_ESM.docx]

**Table 1:** An overview of the disposables used for the preparation and administration of immune checkpoint inhibitors via an elastomeric pump (ICI-P) and conventional infusion (ICI-B).

| **Disposables drug preparation (per item)** | | |
| --- | --- | --- |
| **Type** | **ICI-B** | **ICI-P** |
| Intermate SV 100 105mL (Elastomeric pump) | - | 1 |
| Infusion bag sodium chloride 0.9% 50/100 mL | 1 | 1 |
| Syringe 50 mL | 1 | 2 |
| Blunt fill needle | 2 | 2 |
| Alcohol wipe (Prosat) | 1 | 1 |
| Isopropylalcohol wipe | 4 | 4 |
| Non-woven compress (sterile) 10x10 | 1 | 1 |
| Sterile pair of gloves | 1 | 1 |
| Mask | 1 | 1 |
| Hat | 1 | 1 |
| Shoe-covers | 1 | 1 |
| Minigrip bag | 1 | 1 |
| **Disposables drug administration (per item)** | | |
| **Type** | **ICI-B** | **ICI-P** |
| Chlorhexidine tincture | 0.02 | 0.02 |
| Connection set | 1 | - |
| Adhesive bandages | 0.02 | 0.02 |
| Face mask | 1 | 1 |
| I.V. administration set (ProSet Infusomat) | 1 | - |
| Infusion bag sodium chloride 0.9% 250 mL | 1 | - |
| Infusion filter | 1 (Nivolumab only) | - |
| IV Cannula | 1 | 1 |
| Nonsterile gauze (5x5) | 5 | 5 |
| Nonsterile gloves | 1 | 1 |
| Adhesive Cannula dressing | 1 | 1 |
| Transfersystem with spike adapter | 1 | - |
| Ready-to-administer NaCl flush (10 mL) | - | 1 |
